# Substantial modification of the gene expression profile following exposure of macrophages to welding-related nanoparticles

**DOI:** 10.1038/s41598-018-26988-z

**Published:** 2018-06-04

**Authors:** Étienne Audureau, Angélique Simon-Deckers, Marie-Laure Franco-Montoya, Balasubramanyam Annangi, Ali Kermanizadeh, Jorge Boczkowski, Sophie Lanone

**Affiliations:** 1 Université Paris Est-Créteil, DHU A-TVB, IRMB- EA 7376 CEpiA (Clinical Epidemiology And Ageing Unit), Créteil, F-94010 France; 2 AP-HP, Hôpital Henri-Mondor, Service de Santé Publique, Créteil, F-94010 France; 3 INSERM, U955, Equipe 4, Créteil, F-94000 France; 4 University of Copenhagen, Department of Public Health, Copenhagen, Denmark; 5 Université Paris Est-Créteil, Faculté de Médecine, Créteil, F-94000 France; 6 DHU A-TVB, Service d’explorations fonctionnelles respiratoires, Assistance Publique Hôpitaux de Paris, Hôpitaux Universitaires Henri Mondor, Créteil, F-94000 France

## Abstract

Anthropic nanoparticles (NP) are increasingly produced and emitted, with accompanying concerns for human health. Currently there is no global understanding as to the exact mechanistics of NP toxicity, as the traditional nanotoxicological approaches only provide a restricted overview. To address this issue, we performed an in-depth transcriptomic analysis of human macrophages exposed to a panel of welding-related metal oxide NP that we previously identified in welders lungs (Fe_2_O_3_, Fe_3_O_4_, MnFe_2_O_4_ and CrOOH NP). Utilizing the specified analysis criteria (|fold change| ≥1.5, p ≤ 0.001), a total of 2164 genes were identified to be differentially expressed after THP-1 macrophage exposure to the different NP. Performing Gene Ontology enrichment analysis, for cellular content, biological processes and Swiss-Prot/Protein Information Resource keywords the data show for the first time a profound modification of gene differential expression in response to the different NP, among which MnFe_2_O_4_ NP were the most potent to induce THP-1 macrophage activation. The transcriptomic analysis utilized in the study, provides novel insights into mechanisms that could contribute to NP-induced adverse effects and support the need for widened approaches to supplement existing knowledge of the processes underlying NP toxicity which would have not been possible using traditional nanotoxicological studies.

## Introduction

Anthropic nanoparticles (NP) are increasingly produced and emitted, not only due to their unique desirable properties, but also due to their unintentional release (i.e. during automotive combustion, in tobacco smoke or welding fumes). As such, anthropic NP represent a significant and ever-increasing proportion of particulate atmospheric pollution. Considering the various adverse health effects that have long been associated with atmospheric pollution in the general population as well as in compromised individuals^1–5^, this increasing exposure to anthropic NP is associated with concerns for human health. This is particularly true for carbonaceous NP (i.e. carbon nanotubes and carbon black) as well as metallic NP (i.e. iron, titanium, manganese, chromium oxides), that both highly contribute to anthropic NP pollution^6^.

A large body of data from *in vivo* literature demonstrates that NP can exert adverse effects after pulmonary administration, such as granuloma formation and/or development of lung fibrosis^7^. The specific NP physico-chemical characteristics such as size, elemental composition, surface reactivity, shape, solubility represent major determinants of these potential adverse effects. In general, an inflammatory response, oxidative stress, DNA damages or cell death manifested as necrosis or apoptosis are the most studied mechanisms of toxicity, and as such are classically proposed to be responsible or at least contribute to NP-mediated adverse effects^7^. However, it is also generally acknowledged that the exact mechanistic of NP toxicity is yet not fully elucidated, partly because traditional nanotoxicological approaches only provide a restricted view of what occurs after NP exposure. In the last few years, transcriptomic analysis has allowed for a more comprehensive insight into how altered expression of genetic variants can contribute to toxicological outcomes and complex diseases. Yet, the majority of such studies rapidly focus their analysis on the classical pathways proposed to underlie NP toxicity; immune/inflammatory response, oxidative stress, cell viability, or apoptosis^8–13^. As the single contribution of all or part of these pathways can not explain NP toxicity as a whole, it appears obvious that researchers need to widen the scope of such transcriptomic analysis to identify consistent, prominent and/or novel differentially expressed genes or functional-related gene groups and therefore provide better insights into the mechanisms that could contribute to NP adverse effects.

To achieve this aim, we took advantage of two recent studies performed by our research team: the first one demonstrated that exposure of human macrophages to four different metal oxide NP representative of those identified in the pulmonary tissue of welders (Fe_2_O_3_, Fe_3_O_4_, MnFe_2_O_4_ and CrOOH NP) induces the production of a pro-inflammatory secretome, together with increasing the migration capacity of these cells (except in response to Fe_3_O_4_)^14^. In a concurrent study, we also demonstrated that repeated pulmonary exposure to the same NP was able to induce lung remodeling^15^. The present study was therefore dedicated to better understand the mechanistic pathways involved in the adverse effects observed above, using a transcriptomic approach in NP-exposed human macrophages.

## Results

### Overview of mRNA microarray profiles

**Figure 1:**
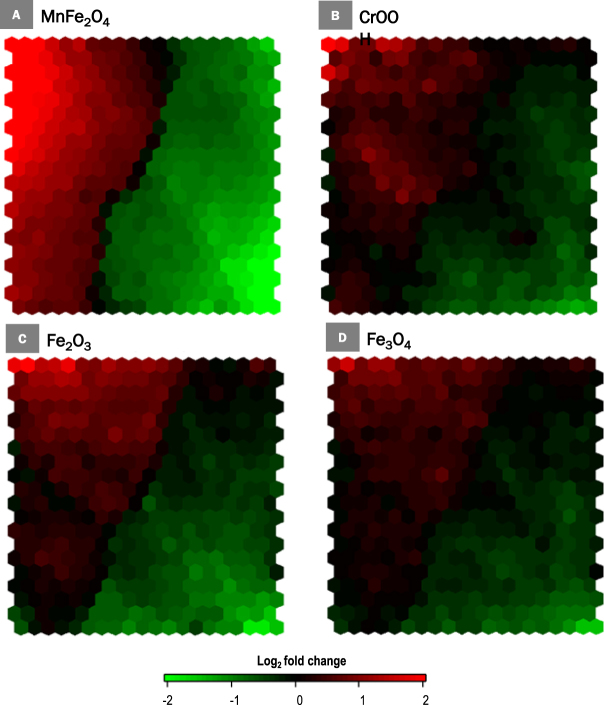
Self-organizing map of differentially expressed genes (N = 2164). Self-organizing map of differentially expressed genes based on fold-changes and shown by nanoparticle: (**A**) MnFe_2_O_4_, (**B**) CrOOH, (**C**) Fe_2_O_3_ and (**D**) Fe_3_O_4_. Each cell of the map represents a set of genes with overall similar differential gene expression and increasing distance between cells indicates increasingly different expression profiles.

**Figure 2:**
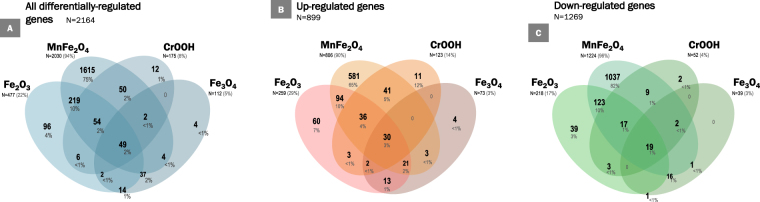
Venn diagrams of differentially expressed genes (N = 2164). Venn diagram of unique and shared genes among the four nanoparticles: (**A**) All differentially expressed genes, (**B**) Up-regulated genes and (**C**) Down-regulated genes. Abbreviations as in Fig. 1.

Utilizing the specified analysis criteria (|FC| ≥1.5, p ≤ 0.001), a total of 2164 genes were found to be differentially expressed after THP-1 macrophage exposure to Fe_2_O_3_, Fe_3_O_4_, MnFe_2_O_4_ or CrOOH NP (Figs 1 and 2A). Exposure to MnFe_2_O_4_ NP induced the highest number of genes differentially expressed (2030, Fig. 2A), as illustrated by the SOM analysis showing more intense expression levels for this NP in most areas of the map, whether indicating up- or down-regulation (Fig. 1). Most of these genes (1615, representing 75% of total number) were unique to MnFe_2_O_4_ NP. Interestingly, only 49 differentially expressed genes were common to all four NP. The majority of the differentially expressed genes was downregulated (1269 downregulated genes as opposed to 899 that were upregulated, Fig. 2B and C), mainly as a result of MnFe_2_O_4_ NP effect: out of the 1269 downregulated genes, 1037 were unique to MnFe_2_O_4_ NP, and a total of 1224 genes were differentially expressed (downregulated) in response to these NP. Exposure to the other 3 NP mainly resulted in the upregulation of genes, particularly in response to Fe_3_O_4_ or CrOOH NP; respectively 65.2% (73 out of 112 genes) and 70.3% (123 out of 175 genes).

**Figure 3:**
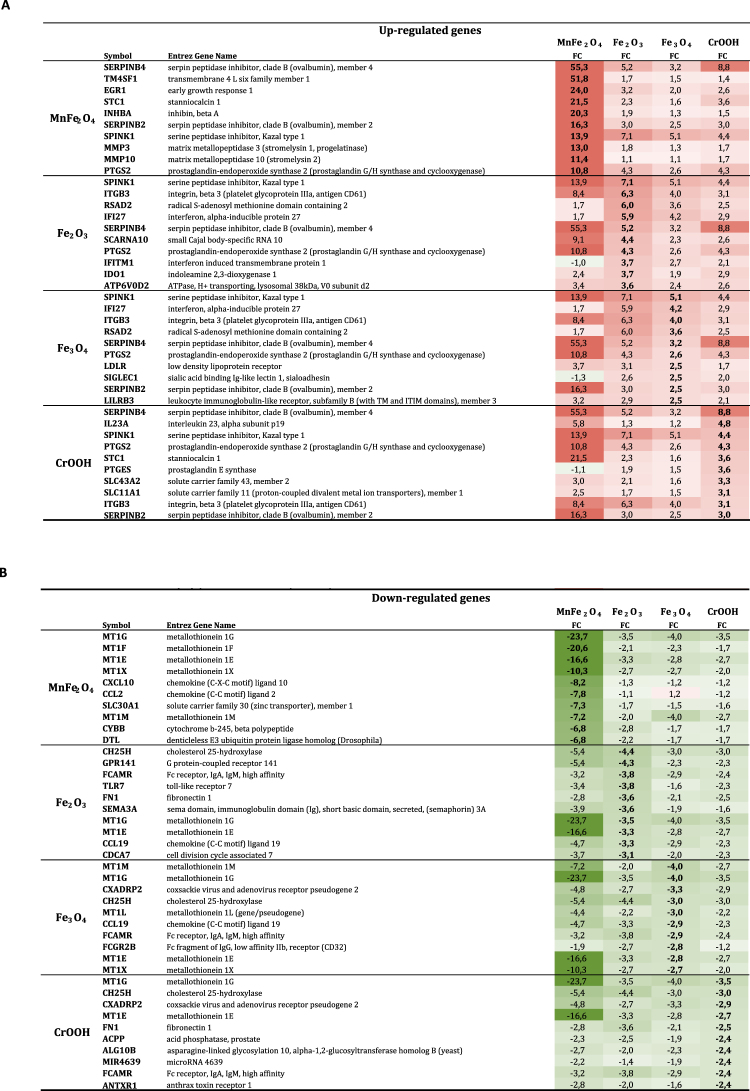
List of the 10 top up-regulated genes (fold-change in red, Panel A) and the 10 top down-regulated genes (fold-change in green, Panel B) by nanoparticle. Abbreviations as in Fig. 1.

The analysis of the top 10 up and down-regulated genes (Fig. 3 and Supplementary Fig. 1) revealed that the amplitude of expression modulation was the highest for MnFe_2_O_4_ NP: the fold change values varied from x55.3 to x10.6 for the top 10 upregulated genes, and from x(−23.7) to x(−6.6) for the top 10 downregulated genes after exposure of the macrophages to these NP. The amplitude of fold change variation after exposure to one of the other 3 NP was much lower, and generally similar for all 3 NP, with a maximum at x8.8 for upregulated genes and x(−4.4) for the downregulated ones. Five of the top 10 up- or downregulated genes were common to all 4 NP: Serpin peptidase inhibitor B4 (Serpin B4), Serine peptidase inhibitor K1 (SPINK1), Prostaglandin-endoperoxide synthase 2 (PTGS2) were upregulated, and Metallothionein 1G and 1E (MT1G and MT1E respectively) were downregulated. Moreover, 9 of the top 10 regulated genes (4 upregulated and 5 downregulated) were unique to MnFe_2_O_4_ NP (fold change lower than 2.5 in response to the other NP), while only 2 of the top 10 regulated gene (1 upregulated and 1 downregulated) was unique to CrOOH. None of the top 10 regulated genes in response to Fe_2_O_3_ or Fe_3_O_4_ NP was specific to either of these NP. Interestingly, 2 genes (Radical S-adenosyl methionine domain containing 2 and Interferon, alpha-inducible protein 27) were present in the top 10 list of Fe_2_O_3_ or Fe_3_O_4_ NP, and significantly modulated by CrOOH NP, but not in response to MnFe_2_O_4_ NP.

### Gene functional analysis

**Figure 4:**
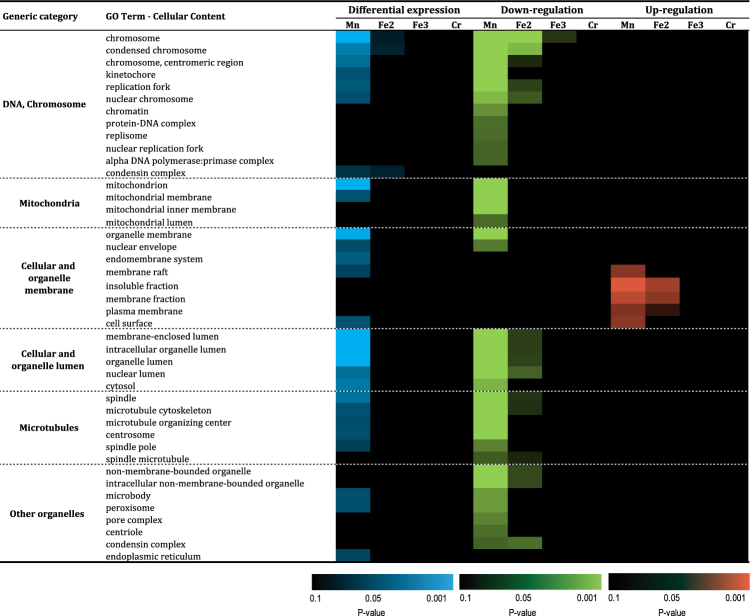
Functional enrichment of significantly regulated genes: cellular content GO-terms. Abbreviations as in Fig. 1.

We further focused on the 2164 differentially expressed genes after THP-1 macrophage exposure to Fe_2_O_3_, Fe_3_O_4_, MnFe_2_O_4_ or CrOOH NP, by performing gene enrichment analysis, for cellular content (CC), biological processes (BP) and Swiss-Prot/PIR keywords. Over-representation of GO cellular contents revealed several common GO-terms including those related to DNA, mitochondria, cellular/organelle membrane, cellular/organelle lumen, microtubules and other organelles (Fig. 4). Interestingly, most of these differentially expressed genes were down-regulated: as shown in Fig. 4, only a handful of functional gene groups relating to cellular and organelle membrane were upregulated (more specifically those related to cellular membrane including membrane raft, plasma membrane and cell surface), while those related to organelle membrane and nuclear envelope were upregulated. These common GO-terms were identified in response to exposure to MnFe_2_O_4_ NP (35 downregulated and 5 upregulated ones), and to a lesser extent following exposure to Fe_2_O_3_ NP (15 downregulated CC GO-term, and 3 upregulated). Only 1 CC GO-term was differentially expressed in response to Fe_3_O_4_ NP (“chromosome”, downregulated), while no GO-term modification was noted in response to CrOOH NP exposure. Overall, the differential expression analysis of up- and downregulated CC terms demonstrate a trend towards the downregulation of cellular membrane content.

**Figure 5:**
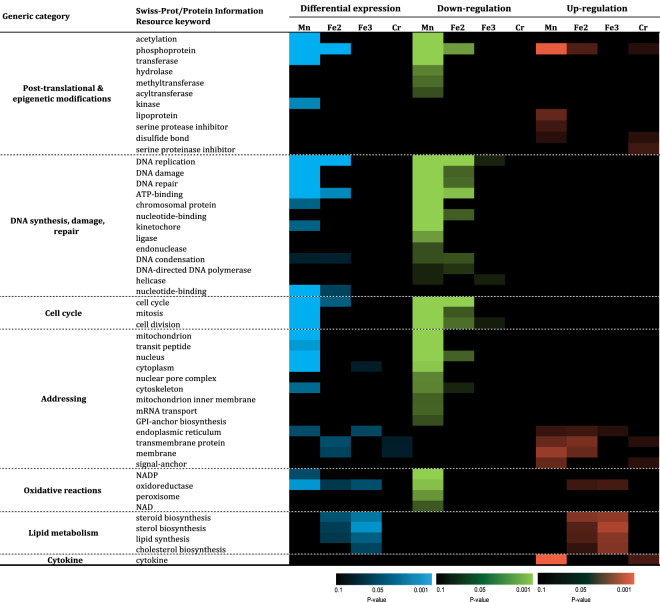
Functional enrichment of significantly regulated genes: Swiss-Prot/Protein Information Resource keywords. Abbreviations as in Fig. 1.

Next, protein enrichment analysis was performed for Swiss-Prot/PIR content on the 2164 differentially expressed genes (Fig. 5). The global analysis revealed that genes related to 7 different categories were differentially expressed which included post-translational & epigenetic modifications, DNA synthesis, damage & repair, cell cycle, addressing, Oxidative reactions, lipid metabolism and cytokine. Most of these differential modulations were observed in response to MnFe_2_O_4_ NP (44 out of 49 significant Swiss-Prot/PIR keywords). However, those related to lipid metabolism was only observed in response to Fe_2_O_3_, Fe_3_O_4_ NP. As for CC GO-terms, most of these functional groups of genes were downregulated (34 downregulated as compared to 15 that were upregulated). Remarkably, 2 categories of genes (related to DNA synthesis, damage & repair, as well as cell cycle) were found to be only downregulated, whereas 2 other categories were up regulated (lipid metabolism and cytokine). Some categories of genes, such as that related to addressing appeared both down- and up regulated, mainly in response to MnFe_2_O_4_ NP but also to the other three NP. Interestingly, and in accordance with CC GO-terms, categories of genes regarding extracellular membrane addressing were upregulated, while those regarding intracellular structures such as mitochondria, nucleus, cytoplasm were downregulated. The exposure to CrOOH NP induced the differential expression of only a few Swiss-Prot/PIR keywords, but these genes were always upregulated (phosphoprotein, disulfide bond, serine proteinase inhibitor, transmembrane protein, signal anchor, and cytokine). Interestingly, lipid metabolism-related genes were specifically upregulated only in response to Fe_2_O_3_ and Fe_3_O_4_ NP.

**Figure 6:**
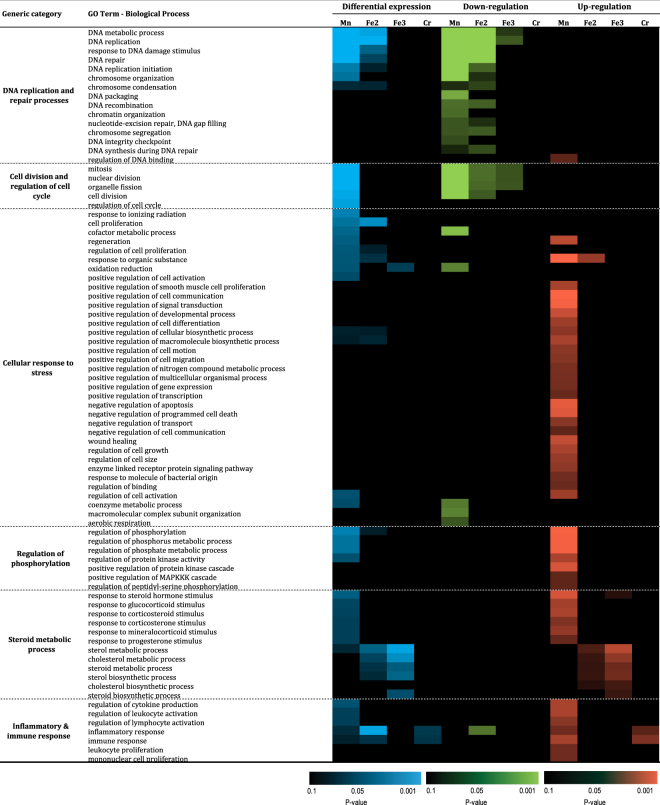
Functional enrichment of significantly regulated genes: biological process GO-terms. Abbreviations as in Fig. 1.

Finally, the analysis was focused on the GO-terms biological processes (BP) among the 2164 differentially expressed genes (Fig. 6). Eighty-one different BP were differentially expressed, and could be attributed to 6 different categories: DNA replication and repair processes, cell division and regulation of cell cycle, cellular responses to stress, regulation of phosphorylation, steroid metabolic processes, and inflammatory & immune responses. As observed for other GO-term profiles, exposure to MnFe_2_O_4_ NP was the more potent to induce differential expression of genes (40 BP), while exposure to Fe_2_O_3_, Fe_3_O_4_ or CrOOH NP lead to the differential expression of 19, 6, and 2 clusters of genes respectively. Four clusters of genes related to the Steroid metabolic process family were only differentially expressed in response to Fe_2_O_3_, or Fe_3_O_4_ NP (cholesterol metabolic process, steroid metabolic process, sterol biosynthetic process and steroid biosynthetic process). As opposed to the other terms analyzed, the overall BP differential expression was upregulated mainly in response to MnFe_2_O_4_ NP exposure (53 GO terms as compared to 24 downregulated). Amongst these, two families of genes were mostly (DNA replication and repair process) or exclusively (cell division and regulation of cell cycle) downregulated while 4 others were predominantly (cellular response to stress, and inflammatory & immune response) or exclusively (regulation of phosphorylation and steroid metabolic process) upregulated. The overall differential regulations are in accordance to findings in terms of CC and/or Swiss-Prot/PIR (DNA synthesis, damage & repair, cell cycle, posttranslational & epigenetic modifications). Interestingly, clusters of genes related to lipid metabolism were upregulated in response to Fe_2_O_3_, and Fe_3_O_4_ NP but not to MnFe_2_O_4_ NP. Finally, the only family of gene that was differentially expressed in response to CrOOH NP exposure were inflammatory & immune response genes.

## Discussion

This is the first study of its kind to compare the profound modification of gene differential expression in human THP-1 macrophages exposed to four different welding-related metal NP. Here, transcriptomic analysis was used to gain a better understanding of the mechanisms and biological pathways affected following NP exposure. This comprehensive analysis would have not been possible with more traditional testing methodologies.

As compared to the three other NP studied in this work, MnFe_2_O_4_ NP was the most potent in inducing THP-1 macrophage activation, in terms of changes in gene expression (number of genes affected, activated pathways, fold change, as well as level of significance of the differentially expressed genes). The findings here are in accordance with two recent studies from our group, using the same batch of welding-representative NP which demonstrated that *in vitro* exposure to MnFe_2_O_4_ NP was more potent in the induction and the production of a pro-inflammatory secretome by THP-1 macrophages in comparison to Fe_2_O_3_ or Fe_3_O_4_ NPs^14^. Similarly, a 3-months repeated exposure regime of the MnFe_2_O_4_ NP resulted in lung remodeling in mice^15^. It is interesting to note that out of the 2030 differentially-regulated genes in response to MnFe_2_O_4_ NP, 1615 were unique to this NP alone (79.6% of all MnFe_2_O_4_ NP differentially-regulated genes), while only 359 (17.7%) were common with Fe_2_O_3_ NP, and 92 (4.5%) with Fe_3_O_4_ NP. Moreover, 49 (2.4%) were common to all Fe-containing NP. This suggests that Mn but not Fe is the chemical element that is mostly driving the macrophagic biological response to MnFe_2_O_4_ NP. Within the iron only NP, Fe_2_O_3_ NP induced the strongest biological response. Indeed, a higher number of GO-terms were significantly modulated in response to Fe_2_O_3_ NP as compared to Fe_3_O_4_ NP, while all but a few of the GO-term differentially modulated in response to Fe_3_O_4_ NP were also modulated in response to Fe_2_O_3_ NP. This is in accordance with our previous *in vitro* study focusing on THP-1 inflammatory response to the same NP^14^. However, this is in slight disagreement with our companion *in vivo* study in mice, where Fe_2_O_3_ NP where overall less reactive than Fe_3_O_4_ NP^15^.

It is difficult to compare this study to others from the literature regarding welding-related NP toxicity. Indeed, transcriptomic studies are relatively new, and if a few of them have been performed after exposure to total welding fumes^16–18^, none have been performed so far focusing on the particular effects of welding-related NP. However, a few studies on cellular response (macrophages, hepatic cells, whole mice) following exposure to various formulations of iron oxide NP (mainly superparamagnetic iron oxide, pristine or coated Fe_2_O_3_ or Fe_3_O_4_ NP) do exist^10,12,19,20^. As an example, Teeguarden and colleagues demonstrated a good correlation between gene transcription profiles obtained in the lungs of mice and that of macrophages *in vitro* after exposure to 12.8 nm superparamagnetic iron oxide particles (SPIO)^12^. Nevertheless, the Teeguarden study utilized a relatively short exposure period of 7 days to identify the acute inflammatory response, whereas we were more interested in the long-term lung remodeling effects only observable following long-term exposure to NPs^14^. The disparity between the studies could be explained by the potential inhibitory effect of soluble iron oxide on inflammation^21^. However, previous data from our lab using the same metal NP, as those used in the present study showed very little or no solubility^14^. A similar discrepancy with our previous *in vivo* findings can be highlighted in the present study: indeed, CrOOH NP did not appear to be strong inducers of any particular biological response, except for the differential expression of “inflammatory & immune response” BP and “cytokine” Swiss-Prot keyword, both leading to the upregulation of the respective GO-term pathways. Overall, this suggests that, beyond the importance of chemical speciation, macrophages might not be the essential or at least not the only target cell type to drive long-term lung remodeling in response to welding-related iron oxide and CrOOH NP. Moreover, we are completely aware that welding fumes cannot be summarized to only metal oxide NP, but as a complex mixture of particles (micrometric and nanometric) together with a number of potentially toxic gases (e.g. ozone, nitrogen oxide, nitrogen dioxide) generated during the welding process^22^. However, we strongly believe that the relevance of our findings is also supported by the fact that we were recently able to link the presence of NP aggregates/agglomerates in lung tissues samples of arc-welders to the presence of similar fibrotic lesions^14^.

The principal body of previous literature dedicated to transcriptomics mainly focus on GO enrichment analysis of genes related to classical pathways of NP toxicity namely immune/inflammatory response, oxidative stress, cell viability, or apoptosis^8–13^. The thorough GO-term profiling analysis performed in the present study allowed us to describe profound modifications of several terms related not only to the classically described pathways such as inflammation, oxidative reactions, DNA synthesis/damage/repair, but also to address specific cellular contents (membrane, mitochondria, etc.), post-translational or epigenetic modifications (such as regulation of phosphorylation). As an example, we identified a clear up-regulation of BP related to (chole)sterol, steroid metabolic/biosynthesis processes, as well as, the up-regulation of the Swiss-Prot keyword “lipid metabolism” only in response to iron-only NP (Fe_2_O_3_ or Fe_3_O_4_). Although the design of the present study does not allow us to further elaborate on the relevance of these findings *in vivo*, they are in accordance with what was found in rat lungs after repeated exposure to welding fumes^17^, and could be related to the described cardiotoxicity of iron NP^23^. Another interesting finding was the identification of the modulations in the differential expression of Swiss-Prot/PIR keywords related to “addressing”, which principally demonstrated a down-regulation of intracellular addressing signals, whereas signals in favor of addressing to plasma membrane or anchoring were up-regulated. These findings were in line with the observed modulations in the differential expression of cellular contents, where GO-terms related to cellular and organelle lumen as well as organelle and nuclear envelope were down-regulated for example. Interestingly, this could also be linked to the observed modulation of genes involved in cytoskeletal reorganization, such as Trem2 and ENG, two genes recently highlighted in the lungs of rats after recurrent exposure to welding fumes^24^. Finally, it was interesting to note the significant modulation of genes involved in post-translational and epigenetic modifications. The identification of the resulting biological effects of such modifications was beyond the scope of the present study, but it is clear that they could be of major importance given their involvement in (lung) disease progression^25^. Overall, our findings highlight and illustrate the importance of the necessity to go beyond the “classical” pathways that are traditionally investigated in the nanotoxicological context.

It must be highlighted that GO-term analysis requires a minimum number of genes for significance. Due to the fact that the enrichment analysis does not allow for the prioritization of the different functions of the identified genes, a degree caution is needed in the analysis of the generated data. This is well illustrated with the analysis of the “top 10” up- and down-regulated genes. Indeed, although not identified strictly speaking as differentially expressed GO-terms, two biological mechanisms were highly represented in this analysis, and were in large accordance with the phenotype of mice repeatedly exposed to the same NP^15^. The first one is remodeling, with over-expression of several genes implied in protease/anti-protease balance (Serpin B4, Serpin B2, SPINK, MMP3, MMP10, etc.) (Fig. 3), as well as STC1, INHBA and EGR1 (involved in the progression and enhancement of fibrosis)^26^. The second biological mechanism is related to ion transport, with the downregulated expression of ion transporters (metallothionein MT1G, MT1F, MT1E, MT1X, MT1M, SLC30A1, etc.). This downregulation of metallothionein expression could be related to pro-fibrotic response observed after NP exposure^15^ since it has been demonstrated that metallothionein administration protects from carmustine-induced lung fibrosis in rats^27^. Although we did not assess the expression of these “top 10” markers in the lung of our repeatedly-exposed mice, these results are in large accordance with the lung remodeling phenotype we observed in these mice^15^.

## Conclusion

Overall, our data provides novel insights into the possible mechanisms that could contribute to NP-induced adverse effects and highlight the need for a more comprehensive analysis which go beyond the classical pathways currently proposed to underlie NP toxicity.

## Methods

All methods and experimental protocols were carried out in accordance with our institutional guidelines, and all experimental protocols were approved by our local quality committee.

### Metal oxide NP

Four NP representative of welding occupational exposure (Fe_2_O_3_, Fe_3_O_4_, MnFe_2_O_4_, CrOOH) were chemically synthetized in aqueous solution using the micro-waved-assisted sol-gel method and characterized as powder and as suspension in ultrapure water after sonication as previously described^14,15^. Briefly, particle nature was determined by X-Ray diffraction (XRD), and their size and shape were evaluated using the Debye-Scherrer equation on several diffraction peaks, together with an analysis of transmission electron microscopy (TEM) images. All NP were spherical and presented an average diameter of 25 nm (Fe_2_O_3_, Fe_3_O_4_, MnFe_2_O_4_) or 15 nm (CrOOH). Stock solutions of NP suspensions (2 mg/mL) were prepared in ultrapure water and stored at −80 °C. Just before use, the suspensions were sonicated for 10 min in ultrasonic bath (Elmasonic S30H).

### Cell culture and nanoparticle treatment

Human monocytic THP-1 cells (ATCC TIB-202) were purchased from ATCC (LGC Standards, Molsheim, France) and cultured in DMEM medium (supplemented with 10% fetal calf serum (FCS) and 100 U/ml Penicillin/Streptomycin). Prior to exposure, the cells were seeded a concentration of 2.5 × 10^5^ cells/cm^2^ in DMEM medium with 30 ng/ml of PMA (Sigma-Aldrich, France) to induce macrophage differentiation for 24 hr^28,29^. The cells were then washed 3x with serum-free DMEM medium and exposed to NP (25 µg/cm^2^, non-cytotoxic, data not shown^14^) for 24 hr. Following exposure, the cells were washed before being harvested for RNA isolation.

### RNA isolation and reverse transcription

The cellular RNA was isolated per manufacturer’s instructions (Qiagen, UK). Briefly, 600 µl of RLT buffer was added to all wells. The cells were homogenised utilising a 26 gauge needle. The cellular components were re-suspended with 600 µl of 70% ethanol. The samples were transferred to RNeasy spin columns and centrifuged for 15 seconds at 8000 g. The flow through was discarded and the columns washed with RW1 and centrifuged for 15 seconds at 8000 g before the flow through was discarded. The DNA in the samples was eliminated by the addition of 80 µl of DNase solution and incubation at room temperature for 15 min. The samples were washed before the addition of 500 µl of RPE buffer to the RNeasy spin column. The samples were centrifuged for 2 min at 8000 g. The RNeasy spin columns were placed inside a fresh 1.5 ml collection tube and 50 µl of RNase free water was added. The collected RNA stored at −80 °C until use.

After validation of the RNA quality with Bioanalyzer 2100 (RNA6000 nano chip kit - Agilent), 50 ng of total RNA was reverse transcribed (Ovation PicoSL WTA System V2 - Nugen). The resulting double strand cDNA was used for amplification based on SPIA technology. The purification was followed by the addition of 3.6 µg of Sens Target DNA which are fragmented and biotin labelled (Encore Biotin Module kit - Nugen). The cDNA was fragmented using a Bioanalyzer 2100, before hybridization to a GeneChip® Human Gene 2.0 ST array (Affymetrix) at 45 °C for 17 hr. The chips were then washed on the GeneChip® fluidic station FS450 following specific protocols (Affymetrix) and scanned using the GCS3000 7 G system. The scanned images were analyzed with Expression Console software (Affymetrix) to obtain the raw data (CEL files) and metrics for Quality Controls.

The expression of some genes identified by microarray was quantified by Q-PCR, as previously described^14,15^.

### Bioinformatics and gene functional analysis

Raw intensity data were normalized by Robust Multi-Array Analysis (RMA) summarization. Potential experimental biases and outliers were detected and accounted for based on data from principal component analysis and unsupervised agglomerative hierarchical clustering using Euclidean distance metric and Ward’s linkage approach. Fold-changes were calculated based on the mean values of the gene expression levels of three biological replicates. Normalized data were analysed by one-way ANOVA for global comparison between experimental NP groups and control, followed by post-hoc Fisher’s least significant difference t-tests for pairwise comparisons between groups. Selection of significant genes used the following filtering criteria: a p-value ≤ 0.001 and an absolute fold-change (FC) of 1.5 or more for each NP group compared to controls.

Lists of selected genes were used to build Venn diagrams describing overlapping differentially expressed genes across NP groups, and mutually exclusive genes specific to a NP, globally and after stratifying between up- (FC ≥ 1.5) and down-regulated (FC ≤ −1.5) genes. Kohonen’s self-organized maps (SOM) were computed for illustrative purpose in order to cluster genes based on values of fold-changes and describe contrasted patterns across NP groups in differentially expressed genes. Briefly, the SOM is a neural network approach useful for analyzing and representing high-dimensional data on two-dimensional grids (“maps”). SOMs were built based on all gene expression data and then plotted stratified by NP, where each cell represents a set of genes with overall similar differential gene expression and increasing distance between cells indicates increasingly different expression profiles.

To further investigate the significance of the differential gene expression patterns across NPs, functional enrichment analysis was performed using DAVID v6.7 functional annotation tool (Database for Annotation, Visualization and Integrated Discovery^30,31^). The analyses included enriched Gene Ontology (GO) functions relating to biological processes (BP) and cellular content (CC), and Swiss-Prot/Protein Information Resource (PIR) keywords (SP_PIR_KEYWORDS) for protein annotation, using a modified Fisher Exact P-Value (the EASE score) was calculated in DAVID for gene-enrichment analysis, considering statistically significant p-values less than 0.01 after Benjamini-Hochberg correction. All the computations were conducted using R 3.3.0 (R Foundation, Austria) and Stata v14.2 (StataCorp, College Station, TX, USA).

## Electronic supplementary material

Supplementary Figure
