# Supplementary material for: Substantial modification of the gene expression profile following exposure of macrophages to welding-related nanoparticles
Source: Sci Rep. 2018 Jun 4;8:8554. doi: 10.1038/s41598-018-26988-z (PMC5986907; doi:10.1038/s41598-018-26988-z)
Supplement: Supplementary file 1 — Supplementary Figure [file 41598_2018_26988_MOESM1_ESM.docx]

Substantial modification of the gene expression profile following exposure of macrophages to welding-related nanoparticles

Étienne AUDUREAU^1,2^, Angélique SIMON-DECKERS^3^, Marie-Laure FRANCO-MONTOYA^3^, Balasubramanyam ANNANGI^3^, Ali KERMANIZADEH^3,4^, Jorge BOCZKOWSKI^3,5,6^, Sophie LANONE^3,5,*^

^1^Université Paris Est-Créteil, DHU A-TVB, IRMB- EA 7376 CEpiA (Clinical Epidemiology And Ageing Unit), Créteil, F-94010, France.

^2^AP-HP, Hôpital Henri-Mondor, Service de Santé Publique, Créteil, F-94010, France.

^3^INSERM, U955, Equipe 4, Créteil, F-94000, France.

^4^ University of Copenhagen, Department of Public Health, Copenhagen, Denmark

^5^Université Paris Est-Créteil, Faculté de Médecine, Créteil, F-94000, France.

^6^DHU A-TVB, Service d'explorations fonctionnelles respiratoires, Assistance Publique Hôpitaux de Paris, Hôpitaux Universitaires Henri Mondor, Créteil, F-94000, France.

***: corresponding author**

Phone and Fax: +33 1 49 81 37 25

Address: Faculté de Médecine de Créteil, 8 rue du Général Sarrail, Créteil, F-94000, France

Email: [sophie.lanone@inserm.fr](mailto:sophie.lanone@inserm.fr)

**Supplementary Figure 1.** Q-PCR quantification of the 5 top up-regulated genes.
